# Ischémie aiguë de membre sévère sanctionnée par désarticulation de sauvetage dans un contexte de syndrome de Nicolau suite à une injection intramusculaire de pénicilline: à propos d’un cas

**DOI:** 10.11604/pamj.2020.37.378.21384

**Published:** 2020-12-24

**Authors:** Somnoma Jean-Baptiste Tougouma, Massiadami Soulama, Issouf Konate, Emmanuel Tapsoba, Nobila Valentin Yameogo, Patrick Dakoure

**Affiliations:** 1 Service de Cardiologie, Centre Hospitalier Universitaire Sourou Sanou, Bobo-Dioulasso, Burkina Faso,; 2 Institut Supérieur des Sciences de la Santé, Université Nazi Boni, Bobo-Dioulasso, Burkina Faso,; 3 Service d'Orthopédie Traumatologie, Centre Hôspitalier Universitaire Sourou Sanou, Bobo-Dioulasso, Burkina Faso,; 4 Service de Dermatologie, Centre Hôspitalier Universitaire Sourou Sanou, Bobo-Dioulasso, Burkina Faso,; 5 Unité de Recherche et de Formation en Sciences de la Santé, Université Joseph Ki-Zerbo, Ouagadougou, Burkina Faso

**Keywords:** Ischémie aiguë de membre, enfant, syndrome de Nicolau, benzathine benzylpénicilline, *case report*, Acute limb ischemia, child, Nicolau syndrome, benzathine penicillin, case report

## Abstract

Nous décrivons le cas d'un enfant de 9 ans qui a présenté une ischémie aiguë sévère de membre dans un contexte de syndrome de Nicolau du membre pelvien gauche faisant suite à une injection intramusculaire glutéale de benzathine-pénicilline. Outre la rareté de l'ischémie aiguë de membre chez l'enfant, la particularité de ce syndrome de Nicolau compliqué associant une thrombose artérielle aiguë et d'une rhabdomyolyse sévère ayant conduit à une désarticulation de sauvetage de la hanche font l'intérêt de ce cas.

## Abstract (en)

We here report the case of a 9-year-old child suffering from Nicolau syndrome involving the left pelvic limb with severe acute limb ischemia following intramuscular benzathine penicillin injection into the buttock. Given the rarity of acute limb ischemia in children, this case of complicated Nicolau syndrome combining acute arterial thrombosis and severe rhabdomyolysis leading to rescue hip dislocation is very interesting.

## Introduction

L´ischémie aiguë de membre est une urgence médicochirurgicale. Elle est très rare chez l´enfant. Le syndrome de Nicolau, ou dermite livedoïde en est une étiologie. Il s´agit d´une complication rare et sévère des injections intramusculaires [1]. Son mécanisme est mal élucidé. On suggère des mécanismes vasculaires prépondérants à type de vasospasme, de thrombose artérielle et d´embolie, un traumatisme direct par l´aiguille d´injection ou une compression péri-vasculaire par le volume injecté [2]. L´évolution est imprévisible avec un risque d´ischémie du membre, une surinfection cutanée, une parésie ou paralysie, des douleurs neuropathiques, ou une rhabdomyolyse parfois fatale. La rareté de l´ischémie aiguë de membre chez l´enfant mais aussi son étiologie font la particularité de ce cas d´un garçon de 9 ans soldé par une désarticulation de sauvetage de la hanche.

## Patient et observation

**Figure 1:**
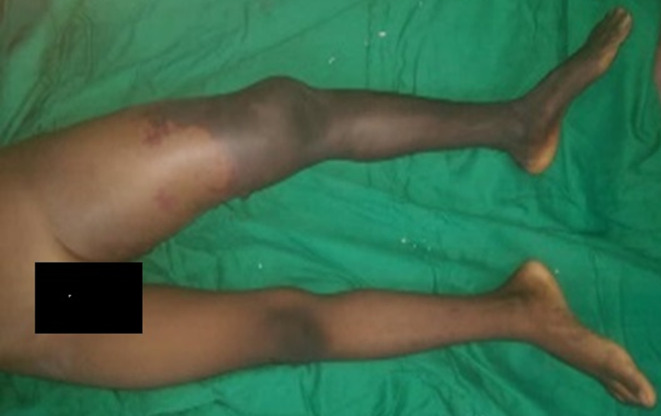
syndrome d'ischémie aiguë du membre pelvien gauche chez un enfant de 9 ans, Bobo-Dioulasso, 2019

L´enfant WZ âgé de 9 ans a été reçu aux urgences cardiovasculaires du Centre Hospitalier Universitaire Sourô Sanou (CHUSS) de Bobo-Dioulasso pour une ischémie aiguë du membre pelvien gauche compliquant une injection intramusculaire de benzatyl benzyle pénicilline (BBP) au niveau de la cuisse gauche effectuée trois jours plus tôt pour le traitement d´une dermatose généralisée. Le patient aurait présenté quelques heures après l´injection, une douleur vive de la cuisse gauche s´accompagnant rapidement d´une impotence fonctionnelle absolue du membre. L´examen clinique notait un général passable; les constantes hémodynamiques étaient normales; il y avait un syndrome d´ischémie aiguë du membre pelvien gauche (Figure 1) (des pétéchies remontant au 1/3 proximal de la cuisse gauche, sécheresse cutanée, refroidissement cutané, insensibilité et momification des 2/3 distaux de la jambe et du pied, une abolition des pouls à l´exception du pouls fémoral qui était perçu).

L´échographie Doppler artérielle des membres pelviens retrouvait une thrombose artérielle totale à partir du 1/3 supérieur de la fémorale superficielle gauche. En amont, un amortissement sévère du flux systolique des artères fémorales superficielle et commune gauche. L´angioscanner notait une très bonne perméabilité vasculaire fémorale gauche jusqu´à 8,5 cm de la bifurcation fémorale au niveau de la fémorale superficielle. Il n´était pas retrouvé de flux vasculaire en dessous de la lésion vasculaire. Par ailleurs, l´angioscanner mettait également en évidence une nécrose extensive des muscles glutéaux avec présence de gaz. Les résultats des explorations biologiques notaient: créatine phosphokinase (CPK) totaux (1031 UI/l dont 1016 UI/l de CPK MM); créatininémie (100,08 umol/l); plaquettes (225000/mm^3^).

**Figure 2:**
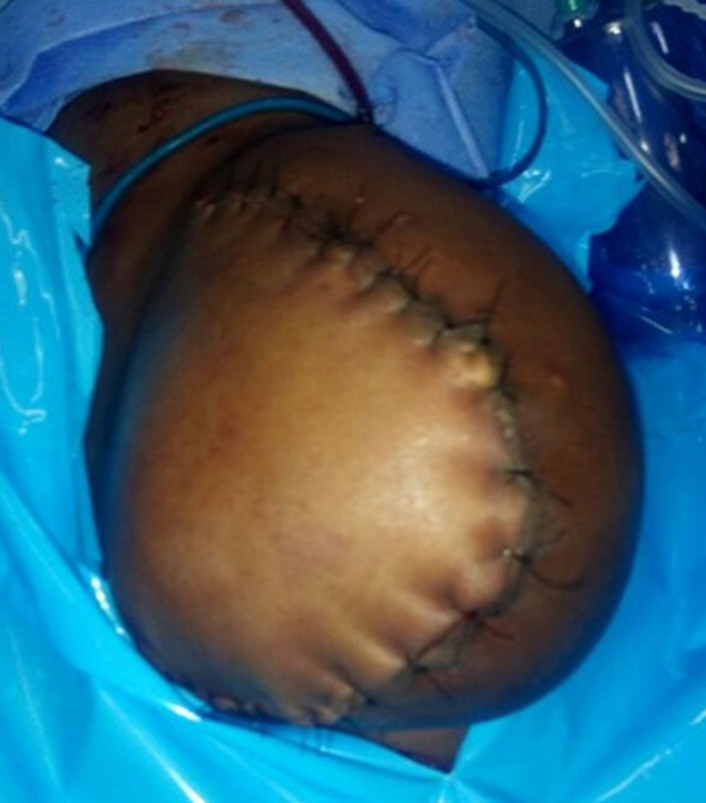
photographie de la hanche gauche après désarticulation chez un enfant de 9 ans, Bobo-Dioulasso, 2019

**Figure 3:**
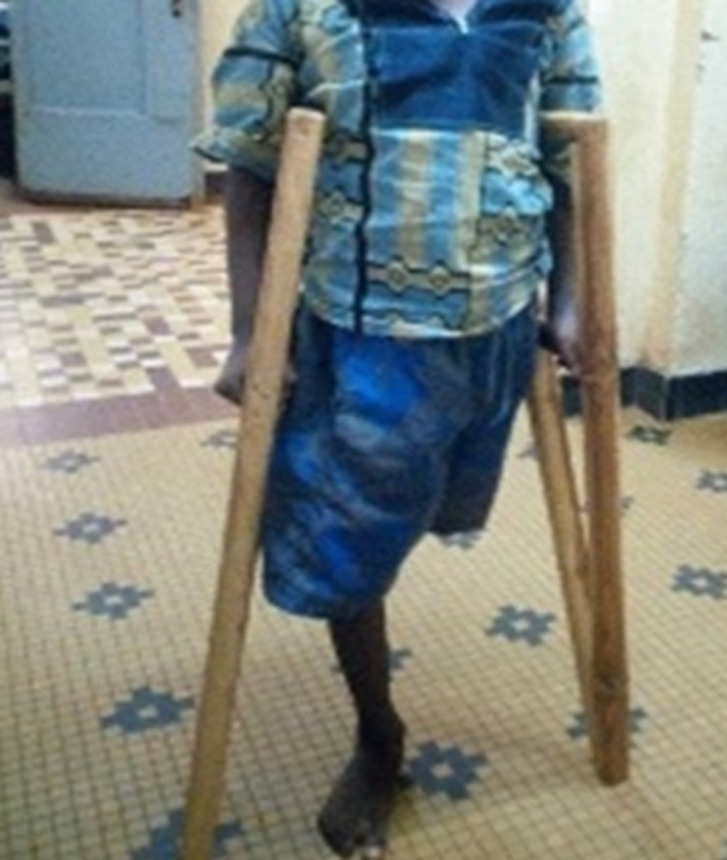
photographie du patient après verticalisation à l'aide d'une paire de béquilles, Bobo-Dioulasso, 2019

Le diagnostic d´un syndrome de Nicolau du membre pelvien gauche compliquant une injection intramusculaire de la cuisse gauche de BBP avec ischémie sévère par thrombose de l´artère fémorale superficielle au 1/3 proximal et une nécrose massive des loges musculaires de la cuisse gauche avec une rhabdomyolyse sévère a été retenu. Il a été indiqué et réalisé une désarticulation de sauvetage de la hanche gauche quatre jours après l´injection (Figure 2). Les suites opératoires ont été simples. Les examens biologiques se sont normalisés (CPK MM à 16 UI/l et Créatininémie à 40,32 umol/l). La cicatrisation du moignon a été obtenue au 14^e^ jour post-opératoire puis la verticalisation et la déambulation du patient ont été effectuées à l´aide d´une paire de béquilles (Figure 3).

## Discussion

L´ischémie aiguë de membre chez l´enfant est une entité rare. L´originalité de notre observation réside dans l´étiologie de cette ischémie. En effet, le syndrome de Nicolau est très rare. Bien plus, la sévérité de l´atteinte vasculaire et musculaire ayant conduit à une désarticulation de sauvetage en fait une particularité. Le syndrome de Nicolau a été décrit pour la première fois en 1924 par Nicolau chez un patient qui avait reçu une injection intramusculaire de sels de bismuth pour une syphilis [3]. Par la suite, plusieurs médicaments ont été associés à ce syndrome [4]. Chez l´enfant, le médicament le plus souvent incriminé est la benzathine-pénicilline, administrée par voie intramusculaire pour l´antibiprophylaxie du rhumatisme articulaire aigu [5,6].

**Figure 4:**
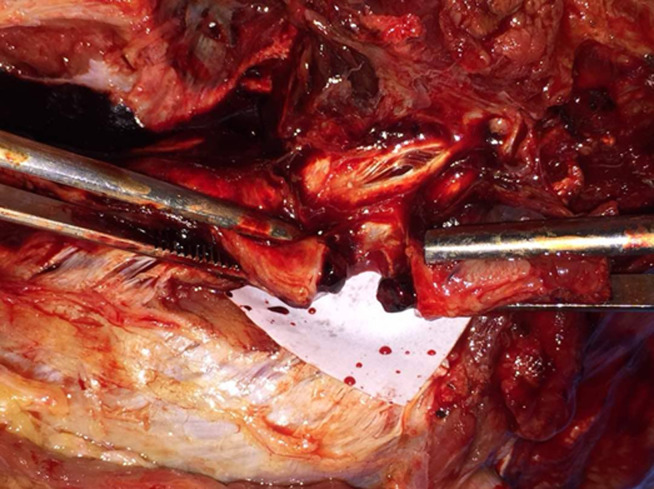
aspect macroscopique de l'artère fémorale superficielle après dissection sur le segment de membre désarticulé montrant l'obstruction de sa lumière par un volumineux thrombus chez un enfant de 9 ans, Bobo-Dioulasso, 2019

Sur le plan physiopathogénique, plusieurs mécanismes avaient été évoqués [2]. Actuellement, l´hypothèse retenue est celle d´un vasospasme artériel aigu aboutissant à une diminution du flux artériel avec risque d´occlusion thromboembolique des vaisseaux de petit calibre, d´ischémie et de nécrose distale [4]. Dans notre cas, l´injection de benzathine-pénicilline avait entraîné une thrombose totale de l´artère fémorale superficielle (Figure 4) par traumatisme direct de l´aiguille d´injection ou par injection directe de la BBP dans l´artère. Le retard au diagnostic et à la prise en charge ont été des facteurs de mauvais pronostic.

En effet, l´enfant nous a été adressé trois jours après le début des symptômes. Un diagnostic précoce aurait certainement permis d´améliorer le pronostic par l´administration en urgence d´anticoagulants et/ou de thrombolytiques. Même si le pronostic vital du patient n´est plus engagé, il reste que la désarticulation de la hanche est une chirurgie lourde de conséquences sur le plan fonctionnel et psychologique. Un soutien psychologique et un appareillage orthopédique permettront certainement de réduire les conséquences du geste chirurgical.

## Conclusion

Le cas rapporté ici, pose le problème de la maîtrise des techniques de soins et des effets néfastes possibles des médicaments. La prévention du syndrome de Nicolau est basée sur le respect des recommandations techniques relatives aux injections intramusculaires, en particulier celles qui préconisent d´aspirer avant d´injecter lentement le produit, et sur l´éviction de toute injection intramusculaire non indispensable, en particulier chez l´enfant.

## References

[1] Elfatoiki FZ, Ennajdi A, Gueddari W, Chiheb S (2017). Nicolau livedoid dermatitis with severe neurological involvement in a child. Ann Dermatol Venereol.

[2] Luton K, Garcia C, Poletti E, Koester G (2006). Nicolau syndrome: three cases and review. Int J Dermatol.

[3] Nicolau S (1925). Dermite livedoide et gangreneuse de la fesse, consecutive aux injections intramusculaires, dans la syphilis: à propos d'un cas d'embolie artérielle bismuthique. Ann Mal Vener.

[4] Alkan Bozkaya T, Demirel G, Ormeci T, Al S, Çakar E, Tastekin A (2016). Anticoagulant and vasodilator therapy for Nicolau syndrome following intramuscular benzathine penicillin injection in a 4 year old boy. Arch Argent Pediatr.

[5] De Sousa R, Dang A, Rataboli P (2008). Nicolau syndrome following intramuscular benzathine penicillin. J Postgrad Med.

[6] Rémond M, Coyle M, Mills J, Maguire G (2016). Approaches to improving adherence to secondary prophylaxis for rheumatic fever and rheumatic heart disease: a literature review with a global perspective. Cardiol Rev.

